# Evaluation of bioaccessibility of bioactive compounds in ready-to-eat refrigerated and frozen broccoli using *in vitro* digestion models

**DOI:** 10.1038/s41598-025-26034-9

**Published:** 2025-11-21

**Authors:** Mohamed Awad Abd Allah, Ghada Khiralla, Hesham Elhariry

**Affiliations:** 1 Department of Medicinal Food, Egyptian Drug Authority (EDA), Known Formerly As National Organization for Drug Control and Research, El Giza, 11221 Egypt; 2 Department of Biotechnology and Food Safety in Arid Land, Arid Land Agricultural Graduate Studies and Research Insitute (ALARI), Ain Shams University, Cairo, Egypt; 3 Department of Food Science, Faculty of Agriculture, Ain Shams University, Cairo, Egypt

**Keywords:** Ready-to-eat food, Bioactive compounds, Antioxidant capacity, Vegetable processing, Nutrient retention, Biochemistry, Biotechnology, Gastroenterology

## Abstract

Refrigerated and frozen ready-to-eat cooked vegetables are becoming increasingly popular with consumers and catering services. Simulating digestion is essential for accurately assessing the nutritional value of ready-to-eat vegetables, as raw composition data alone may overestimate their health benefits. Therefore, this study evaluated the bioaccessibility of bioactive compounds in broccoli after heat treatment, storage, and *in vitro* gastrointestinal digestion. Fresh broccoli (FB) and heat-treated (boiled or steamed) broccoli were subjected to refrigerated (RBB, RSB) or frozen (FBB, FSB) storage. FB exhibited high phenol (610 mg GAE/100 g) and flavonoid (295 mg QE/100 g) contents. Thermal treatment significantly decreased the phenolic content to 503, 515, 368, and 393 mg GAE/100 g in RBB, RSB, FBB, and FSB, respectively. Total phenols, flavonoids, vitamin C, antioxidant capacity, dietary fibers, and phenolic profiles were analyzed before and after *in vitro* gastrointestinal digestion. After *in vitro* gastrointestinal digestion, phenol, flavonoid, and vitamin C contents decreased significantly compared to those after digestion with FB (DFB). No significant changes in total, soluble (SDF), or insoluble (IDF) dietary fiber were observed between heat-treated broccoli and FBs. However, *in vitro*, gastrointestinal digestion of FB decreased SDF from 1.84 to 1.59% and increased IDF from 1.02 to 1.3%. HPLC analysis revealed substantial phenolic compound losses after *in vitro* gastrointestinal digestion, ranging from 64.9% in DFB to 88% in DFBB. After digestion, the recovery of bioactive compounds decreased, particularly for vitamin C and phenolics. These findings emphasize the importance of simulating digestion when evaluating the nutritional value of processed vegetables, as relying solely on raw composition data may overestimate health-promoting compound intake. Therefore, dietary recommendations should consider cooking methods and the loss of bioactive substances during digestion. Further research is needed to gain deeper insights into the bioaccessibility of antioxidant compounds after the digestion of cooked and preserved vegetables.

**Supplementary Information:**

The online version contains supplementary material available at 10.1038/s41598-025-26034-9.

## Introduction

Broccoli (*Brassica oleracea* var. *italica*), a cruciferous vegetable widely consumed across the globe, has emerged as a functional food due to its remarkable composition of bioactive phytochemicals. Alongside cauliflower, kale, and cabbage, broccoli is particularly rich in vitamin C, carotenoids, flavonoids, phenolic acids, glucosinolates, and their hydrolysis products, such as sulforaphane and indole-3-carbinol. These compounds contribute not only to the plant’s antioxidant potential but also to a range of reported health-promoting effects, including anti-inflammatory, antimicrobial, cardioprotective, antidiabetic, and anticancer activities^1–3^. In particular, sulforaphane has received notable attention for its role in modulating cellular detoxification enzymes and redox-sensitive pathways, while indole-3-carbinol has been implicated in the regulation of estrogen metabolism and DNA repair systems^4,5^.

A growing body of epidemiological and clinical evidence underscores the benefits of frequent broccoli consumption. Several studies have linked regular intake of broccoli and other Brassica vegetables to a reduced incidence of colorectal, breast, prostate, bladder, and kidney cancers^6^. Beyond cancer, beneficial effects have been documented in metabolic health, with broccoli compounds shown to improve lipid profiles, glucose regulation, and vascular function^7^. Such outcomes highlight broccoli as an important nutritional strategy in chronic disease prevention and management.

Nevertheless, the health value of broccoli is highly sensitive to post-harvest processing and storage conditions. Domestic cooking methods such as boiling, steaming, and microwaving can significantly alter the concentration and integrity of phytochemicals. Vitamin C and glucosinolates are particularly labile under heat, with boiling causing the greatest losses due to leaching into cooking water, while steaming generally results in better retention^1,8^. Freezing and refrigeration also affect phytochemical stability, with storage time and temperature playing critical roles. Given the expanding reliance on ready-to-eat and minimally processed vegetables in modern diets, these changes in nutrient composition must be carefully assessed to ensure that consumers receive meaningful health benefits.

Despite the importance of composition data, measuring only the nutrient content of raw or processed broccoli does not provide a complete picture of its biological potential^9,10^. Freezing and refrigeration have multifaceted effects on bioactive compounds in vegetables, particularly in broccoli. While freezing can lead to the degradation of sensitive compounds such as vitamin C and certain flavonoids due to ice crystal formation and cellular disruption, it may also enhance the extractability of bound phenolics by breaking down cell walls, thereby increasing their bioaccessibility^11^. For bioactive compounds to exert health effects, they must be released from the food matrix and undergo appropriate transformations during gastrointestinal digestion, which may enhance or reduce their biological activity depending on their stability, metabolic conversion, and subsequent absorption. This has made *in vitro* gastrointestinal digestion (IVGD) models indispensable for modern food and nutrition research. Standardized protocols, such as those proposed by the INFOGEST network^12^, simulate physiological digestion in the mouth, stomach, and small intestine. Another study examined the effect of *in vitro* gastrointestinal digestion, without considering the oral phase^13^, allowing researchers to evaluate the bioaccessibility of phytochemicals^13,14^. 

Findings from IVGD studies consistently indicate that antioxidant-rich vegetables and fruits undergo substantial losses of key compounds during digestion. For example, broccoli vitamin C has been reported to decrease by over 90%, while flavonoids and hydroxycinnamic acids decline by 80–84% during simulated digestion^15,16^. Similar results have been documented in fruits:* in vitro* digestion of pomegranate juices and extracts led to significant reductions in total phenolics and antioxidant activity, despite the release of some ellagitannin-derived metabolites^17^. Berry matrices such as blueberries and strawberries also exhibit marked decreases in anthocyanins and flavonoids, reinforcing the general observation that bioactive concentrations measured in undigested foods may overestimate their actual nutritional contribution.

Recent advances have expanded the scope of IVGD applications. For example, Salas-Millán and Aguayo^14^ tracked the bioaccessibility and transformation of polyphenols, sulforaphane, and indoles in a novel fermented broccoli-leaf beverage, demonstrating significant shifts in antioxidant activity and compound stability. Similarly, García-Pérez et al.^18^ examined *Brassica microgreens* and showed that gastrointestinal digestion and colonic fermentation reshape phenolic profiles and enhance the release of certain metabolites. *In vitro* gastrointestinal digestion may result in an increase of bioactive compounds, or it may have no effect at all^19,20^. Encapsulation technologies have also been tested to improve stability; in a recent study, microencapsulation of broccoli sulforaphane increased its bioaccessibility from ~ 20% to nearly 70% during simulated digestion^21^. Numerous studies corroborate that *in vitro* gastrointestinal digestion markedly lowers the bioactive contents in fruit matrices. For instance, digestion simulations of five common fruit juices reported phenolic reductions ranging from 7.8% in apple to 35% in kiwi, while antioxidant capacity showed mixed results—with some juices paradoxically increasing and others, like kiwi, decreasing by ~ 19%^22^. Furthermore, a broader survey of eight fruit juices (including pomegranate, orange, and grape) revealed that only 13–27% of total phenolics and 24–67% of flavonoids remained bioaccessible post-digestion, with antioxidant activity dropping to less than 10–30% of original levels^23^. Such findings underline the dual role of digestion: while many parent compounds degrade, new metabolites may emerge that continue to contribute to biological activity.

Analyzing the raw composition of foods alone does not reflect their true nutritional value. Although fresh broccoli and fruits are rich in phytochemicals, many of these compounds undergo transformations during cooking, storage, and digestion, which can limit their bioaccessibility. Simulated digestion models are therefore essential for evaluating which bioactive compounds remain available for absorption and can contribute to health benefits. This approach provides a more realistic understanding of the compounds that may actually reach the human body in an active form.

Accordingly, the present study aims to investigate the combined effect of thermal processing (boiling and steaming), storage conditions (refrigeration and freezing), and simulated gastrointestinal digestion on the bioaccessibility of bioactive compounds in broccoli. By analyzing changes in phenolic content, antioxidant capacity, vitamin C, and dietary fiber across all stages, the study seeks to provide a comprehensive understanding of how processing and digestion interact to influence the nutritional value of ready-to-eat vegetables.

## Materials and methods

### Chemicals

All chemicals, standards, and *in vitro* gastrointestinal digestion fluids used in the experiments were purchased from Sigma Aldrich Chemical Co. (St. Louis, MO, USA). Chemicals were of analytical grade except for the eluting solvents, which were of HPLC grade.

### Preparation of broccoli samples

**Fig. 1:**
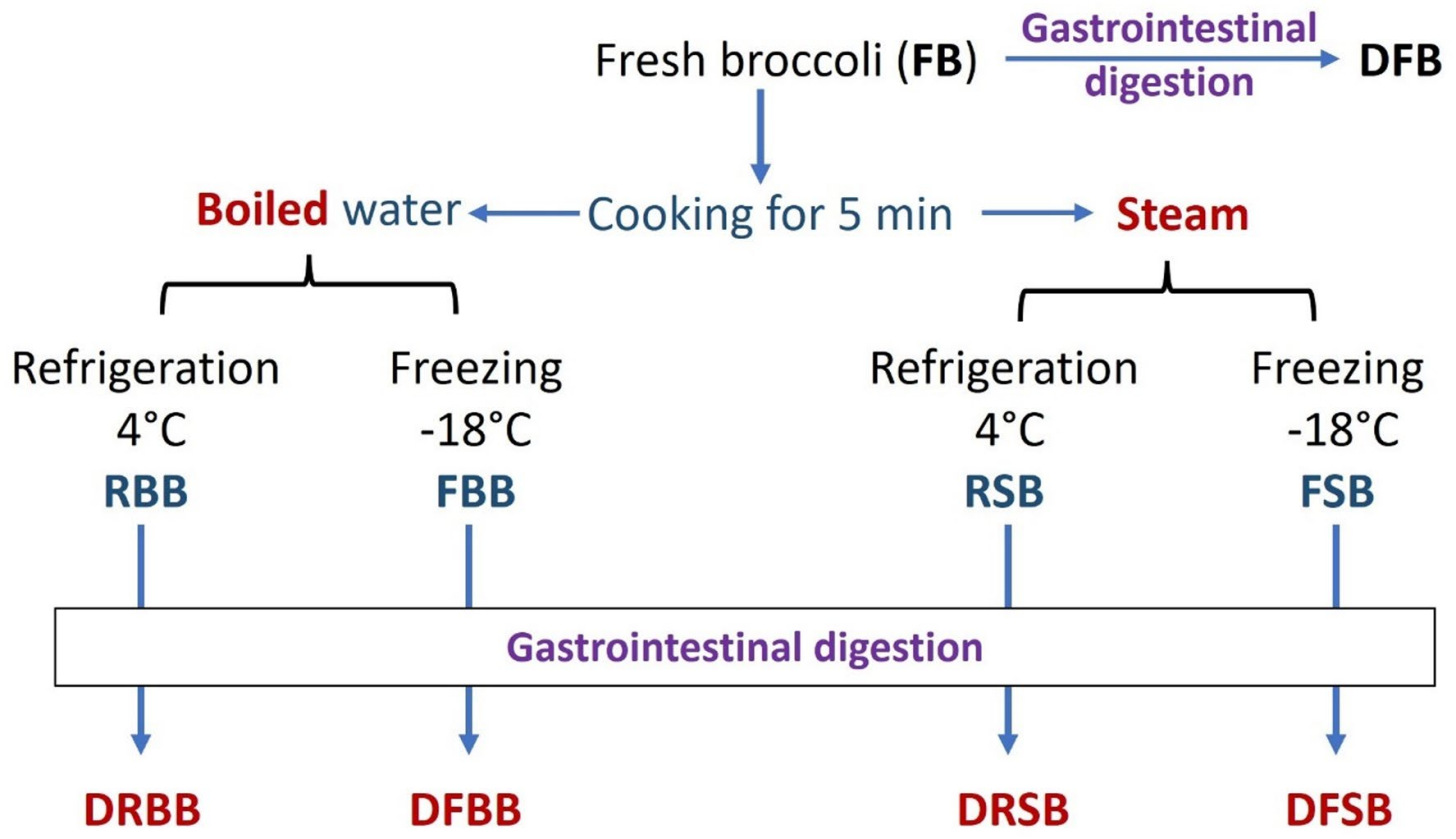
The preparation and gastrointestinal digestion of ready-to-eat cooking of broccoli. *FB* fresh broccoli, *RBB* refrigerated boiled broccoli, *FBB* frozen boiled broccoli, *RSB* refrigerated steamed broccoli, *FSB* frozen steamed broccoli, *DFB* digested fresh broccoli, *DRBB* digested refrigerated boiled broccoli, *DFBB* digested frozen boiled broccoli, *DRSB* digested refrigerated steamed broccoli, *DFSB* digested frozen steamed broccoli.

Fresh broccoli (FB) was obtained from a local market in Giza City, Egypt. Each edible unit was divided into individual inflorescences, washed, dried, and then cut into portions of approximately 100 g. Ready-to-eat broccoli samples were prepared to reflect common domestic practices for ready-to-eat vegetables, and the refrigeration and freezing durations simulate typical consumer and commercial storage scenarios. Ten broccoli samples were prepared as described in Fig. 1. The prepared broccoli was cooked for 5 min in boiling water or in steam^24^. Each cooked broccoli set (boiled and steamed) was divided into two portions. The first sample was stored at 4 °C for 2 weeks and called refrigerated boiled-broccoli (RBB) and refrigerated steamed-broccoli (RSB), while the second sample was stored at − 18 °C for 2 months and called frozen boiled-broccoli (FBB) and frozen steamed-broccoli (FSB). These sets of samples (FB, RBB, RSB, FBB, and FSB) were named undigested samples.

### *In vitro* gastrointestinal digestion of broccoli

The *in vitro* digestion method described by Cassani et al.^25^ was applied in the present study. The test was divided into two stages: gastric digestion using simulated gastric juice and intestinal digestion using simulated intestinal fluid. Ten grams of FB, RBB, RSB, FBB and FSB were homogenized for 10 min with 70 mL of distilled water, and then 10 mL of simulated gastric juice (7.30 g/L NaCl, 0.52 g/L KCl, 3.78 g/L NaHCO_3_, 3 g/L pepsin, at a final pH adjusted to 2.5) was added. The mixture was incubated at 37 °C for 1.5 h under continuous shaking at 100 rpm. To reduce the gastric digestion, the digests were kept in an ice bath for 10 min^12^. Afterwards, 10 mL of simulated intestinal fluid (1.27 g/L NaCl, 0.23 g/L KCl, 0.64 g/L NaHCO_3_, 1 g/L pancreatin, 1.5 g/L bovine bile salts) was added. The final pH was adjusted to 8.0. The resulting mixture was incubated at 37 °C for 3 h under continuous shaking at 100 rpm. To reduce intestinal digestion, the samples were kept in an ice bath for 10 min^12^.

### Sample preparation for analysis

After digestion, the samples were homogenized for 10 min and then centrifuged at 8500 rpm for 20 min. The supernatants were collected and filtered through Whatman No. 1 filter paper. The pellets were collected for analysis of insoluble fibers. All the samples were stored at − 20 °C until analysis. The digested samples were named DFB, DRBB, DRSB, DFBB and DFSB, as described in Fig. 1. For the undigested samples (FB, RBB, RSB, FBB and FSB), 10 g was mixed with 90 mL of distilled water^26^. The mixture was homogenized for 10 min and then centrifuged at 8500 rpm for 20 min. The supernatants were collected and filtered using Whatman No. 1 filter paper. The samples were stored at − 20 °C until analysis.

### Bioactive components

#### Determination of total phenols

Total phenolic content (TPC) was determined before and after *in vitro* gastrointestinal digestion in the supernatant of broccoli samples as described by the Folin–Ciocalteu procedure^27^. Five milliliters of Folin–Ciocalteu reagent (10%) and 4 mL of Na_2_CO_3_ (7.5%) were added to 1 mL of each sample. The mixture was incubated for 5 min at 50 °C. The absorbance was measured at 765 nm. The TPC was calculated using a calibration curve for gallic acid (GA) within the range of 50–400 mg GA/100 mL. The results are expressed as mg gallic acid equivalent (mg GAE)/100 g of dry matter.

#### Determination of total flavonoids

The total flavonoid content (TFC) was determined as described by Lv et al.^28^. One milliliter of each broccoli sample was mixed with 1 mL of 5% sodium nitrite. After 5 min of incubation, 1 mL of 10% aluminum chloride was added to the mixture, and then 10 mL of 4% NaOH was added and incubated for an additional 15 min. Then, the absorbance was measured at 510 nm. The TFC was calculated using quercetin calibration within the range of 20–200 mg/100 mL. The results are expressed as mg of quercetin equivalent (QE) per 100 g of dry matter.

#### Determination of vitamin C

The ascorbic acid (AAC) content was determined by HPLC according to the method of^29^. Samples (20 μL) of supernatant extracted from the different broccoli treatments were analyzed with a Merck-Hitachi (Tokyo, Japan) liquid chromatograph equipped with an L-4000 UV detector and an L-6000 pump. A Zorbax PLRP-S (5 μm, 250 × 4.6 mm) column was used for separation. HPLC elution was carried out at 30 °C, and an isocratic mixture of acetonitrile and 0.05 M KH_2_PO_4_ (70:30, v:v) was used at a flow rate of 0.4 mL/min as the mobile phase. The detection wavelength was 220 nm. The results are expressed as mg ascorbic acid (AA) per 100 g of dry matter.

#### Determination of total dietary fibers

The total dietary fiber (TDF) of the samples was measured according to the method described by A.O.A.C.^30^. The method is based on the gelatinization of the samples and subsequent enzymatic digestion using heat-stable α-amylase, protease and amyloglucosidase. Briefly, in a 400 mL beaker, 1 g of each sample was mixed with 50 mL of phosphate buffer (pH 6.0) and 0.1 mL of α-amylase. The mixture was incubated in a water bath at 90 °C for 15 min with gentle shaking. The pH was adjusted to 7.5 ± 0.1 using NaOH solution (0.275 N). Protease (5 mg) was added to each sample and kept at 60 °C for 30 min with gentle shaking. After cooling to room temperature (approximately 27 °C), the pH was adjusted to 4.5 ± 0.2 using 0.325 M HCl solution. Then, 0.3 mL of amyloglucosidase was added, and the mixture was incubated at 60 °C for 30 min with gentle shaking. After digestion with amyloglucosidase, ethanol (280 mL) was added to precipitate proteins and soluble polysaccharides. After complete precipitation, the content was filtered through a pre-weighed filtered crucible, the residue was washed three times with ethanol (95%) and acetone, and the crucible was dried at 105 °C to a constant weight. After cooling to room temperature, the crucible was weighed. The protein content was determined using Kjeldahl procedures^30^. Another duplicate sample (crucible) was ashed for 5 h at 525 °C, cooled and weighed to the nearest 0.1 mg. Blanks (without sample) were subjected to the same procedure. Total dietary fiber (TDF) was calculated as follows:

1 $$\% {\text{ TDF}} = [{\text{R}} - \left( {{\text{P}} + {\text{A}} + {\text{B}}} \right)/{\text{averagesample weight}}] \times 100$$

where R = average residue weight (mg); P = average protein weight (mg); A = average ash weight (mg); B = average blank weight (R_blank_ − P_blank_ − A_blank_).

#### Determination of insoluble dietary fibers (IDF)

After cisplatin enzymatic digestion as described above for the TDF method, the remaining residue was washed twice with 10 mL of ethanol (95%) and then twice with 10 mL of acetone. The residue was dried at 105 °C to a constant weight, cooled and weighed (D_1_). The dried residue from the dietary fibers was incinerated at 550 °C for at least 5 h, cooled and weighed to the nearest 0.1 mg (I_1_)^31^.

#### Determination of soluble dietary fiber (SDF)

The combined filtrate was adjusted to 100 mL using washing water, and 400 mL of ethanol (95%) was added and allowed to precipitate. The filtrate was then filtered through a dry and weighed crucible containing 0.5 g of Celite and washed twice with 10 mL of acetone. The precipitate was then dried overnight at 105 °C, cooled, and washed (D_2_). The dried material was incinerated at 550 °C for at least 5 h and weighed after cooling (I_2_).

The same procedures were repeated without samples for either insoluble or soluble dietary fiber (B_1_ and B_2_) according to^31^.

Insoluble and soluble dietary fibers were calculated as follows:

2 $$\% \;{\text{IDF}} = \left[ {\left( {{\text{D}}_{1} - {\text{I}}_{1} - {\text{B}}_{1} } \right)/{\text{W}}} \right] \, \times \, 100$$

3 $$\% \;{\text{SDF}} = \left[ {\left( {{\text{D}}_{2} - {\text{I}}_{2} - {\text{B}}_{2} } \right)/{\text{W}}} \right] \, \times \, 100$$

W = weight of sample (g), D = weight after drying (g), I = weight after incineration (g), B = weight of ash-free blank (g).

Following *in vitro* gastrointestinal digestion, the digested samples were centrifuged to separate the pellet (insoluble fraction) from the supernatant (soluble fraction). The pellet was used for insoluble dietary fiber (IDF) determination, following the A.O.A.C.^30^^,^^31^ protocols. The supernatant was used for soluble dietary fiber (SDF) analysis by ethanol precipitation, filtration through Celite, drying, and incineration. Total dietary fiber (TDF) was calculated as the sum of IDF and SDF. All measurements were corrected for blanks and ash content, and fiber percentages were calculated using the standard gravimetric equations.

### Recovery index of bioactive components

The bioaccessibility of the bioactive component (BAC) after heat and *in vitro* digestion treatments was determined by calculating the recovery index (RI) as described by^32^. For each BAC (total phenols, total flavonoids, ascorbic acid, and dietary fiber), the RI was calculated as follows:

4 $${\text{RI}} = \left( {{\text{BAC after treatment}}/{\text{BAC before treatment}}} \right) \times 100$$

### Antioxidant capacity

#### Free radical scavenging capacity

The free radical scavenging activity of the extracts was evaluated by 2,2-diphenyl-2-picryl-hydrazyl (DPPH) according to^33^. Fifty microliters of each broccoli sample were mixed with 1 ml of 0.1 mM DPPH in 80% methanol. After incubating at room temperature for 30 min in the dark, the absorbance of the mixture was measured at *A*_*517nm*_ using a spectrophotometer (Shimadzu model 1601, Japan). The percentage of antiradical activity (ARA) against DPPH was calculated according to the following equation:

5 $${\text{ARA}}\% = \frac{{{\text{A}}_{517nm}{\text{ of control }} - {\text{A}}_{517nm}{\text{ of sample}}}}{{{\text{A}}_{517nm}{\text{ of control}}}} \times 100$$

#### Ferric-reducing antioxidant power (FRAP)

The FRAP assay was conducted according to^34^. Five hundred microliters of the broccoli samples were mixed with 3.6 mL of the FRAP working solution (0.3 M acetate buffer, 0.02 M ferric chloride, 0.01 M 2,4,6-tripyridyl-S-triazine in 0.04 M hydrochloric acid at a ratio of 10:1:1). After the mixture was incubated at 37 °C for 10 min, the absorbance at 593 nm (*A*_*593nm*_) was recorded.

The FRAP was calculated as a percentage (%) using the following equation.

6 $${\text{FRAP value }}\% = \left[ {\left( {As - Ab} \right)/\left( {Ac - Ab} \right)} \right] \times 2$$

*As* = Absorbance of the sample, *Ab* = Absorbance of the blank (distilled water), *Ac* = Absorbance of the positive control (ascorbic acid).

#### Retained activity

The retained activity (%RA) of the scavenging activity, reducing power and total antioxidant activity after treatment (heat treatment and/or gastrointestinal digestion) was calculated as follows:

7 $$\% {\text{ Retained activity}} = \left( {{\text{Activity after treatment}}/{\text{Activity of fresh broccoli}}} \right) \times 100$$

### Comparative analysis of phenolic compounds by HPLC

Identification and quantification of phenolic compounds were performed by an Agilent HPLC system (1260 series, Agilent Technologies, USA) according to^35^. The supernatant of broccoli sample juice was filtered through a 0.45 μm nylon syringe filter and injected into HPLC. A C18 reverse-phase column (250 × 4.6 mm, 5 μm) was used for separation, and the mobile phase consisted of 1% formic acid–water (A) and methanol (B); the mobile phase gradient used was described previously^36^. The mixture was then interfaced with a UV detector (λ = 280 nm) at 25 °C. The phenol content is expressed as μg/100 g on a fresh weight tissue based on the standards of phenolics.

### Statistical analysis

For the combined effect of heat treatment and storage conditions as integrated treatments, data from three independent measurements were collected, and the mean values ± standard deviations (SD) were determined and analyzed by one-way ANOVA using ‘Proc Mixed’ (SAS 8.2, Cary, NC). Differences between treatments were determined by Duncan’s method. In all cases, the level of statistical significance was *P* < *0.05*.

## Results and discussion

### Bioactive components before and after *in vitro* gastrointestinal digestion

The effects of heat treatments and storage conditions on total phenols, flavonoids, and vitamin C in undigested broccoli samples are presented in Table 1. In general, there were notable variations in the concentrations of these bioactive compounds across the different treatments. Fresh broccoli (FB) exhibited the highest levels of total phenols, total flavonoids, and vitamin C among all treatments, with values of 610 mg GAE/100 g, 295 mg QE/100 g, and 139 mg AA/100 g, respectively. The refrigerated broccoli, whether boiled (RBB) or steamed (RSB), showed decreased concentrations of these bioactive components compared to fresh broccoli. Moreover, boiling led to a more significant reduction in total phenols, total flavonoids, and vitamin C than did steaming (Table 1). In general, frozen broccoli, both boiled (FBB) and steamed (FSB), displayed the lowest levels of total phenols, total flavonoids, and vitamin C. Boiling appeared to have a significant (*p* ≤ 0.05) impact on the reduction of these bioactive compounds compared to steaming, regardless of whether the broccoli was refrigerated or frozen (Table 1).

**Table 1:** Effect of heat treatments and storage on total phenols, flavonoids, and vitamin C in ready-to-eat broccoli.

Treatment	Bioactive components*					
	T. Phenols		T. Flavonoids		Vit. C	
	mg GAE/100 g	RI %	mg QE/100 g	RI %	mg AA/100 g	RI %
Fresh broccoli (FB)	610 ± 19^a^	–	295 ± 6^a^	–	139 ± 0.74^a^	–
Refrigerated broccoli						
Boiled (RBB)	515 ± 23^b^	84.4	177 ± 5^c^	60	104 ± 0.51^c^	74.8
Steamed (RSB)	503 ± 6^b^	82.4	198 ± 2^b^	67.1	119 ± 0.66^b^	85.6
Frozen broccoli						
Boiled (FBB)	368 ± 11^cd^	60.3	132 ± 5^d^	44.7	102 ± 0.22^c^	73.4
Steamed (FSB)	393 ± 8^c^	64.4	141 ± 51^d^	47.7	113 ± 0.35^bc^	81.3

*Values are presented as the means (*n* = 3) ± SDs. Values with the same subscribed letters in the same column indicate nonsignificant differences (*p* < 0.05).

Freezing leads to a greater reduction in phenols and flavonoid contents compared to refrigeration due to the formation of ice crystals that rupture plant cell walls, causing enzymatic and oxidative degradation during thawing and cooking. This structural damage facilitates the leaching of phenols, flavonoids, and other bioactive compounds, making them more susceptible to loss. In contrast, refrigeration preserves cellular integrity, minimizing enzymatic activity and oxidation, which helps retain more flavonoids after cooking^10,37^. This explains why the RI of phenols and flavonoids is significantly higher in refrigerated samples (82.4–84.4% and 60–67.1%, respectively) than in frozen ones (60.3–64.4% and 44.7–47.7%, respectively).

**Fig. 2:**
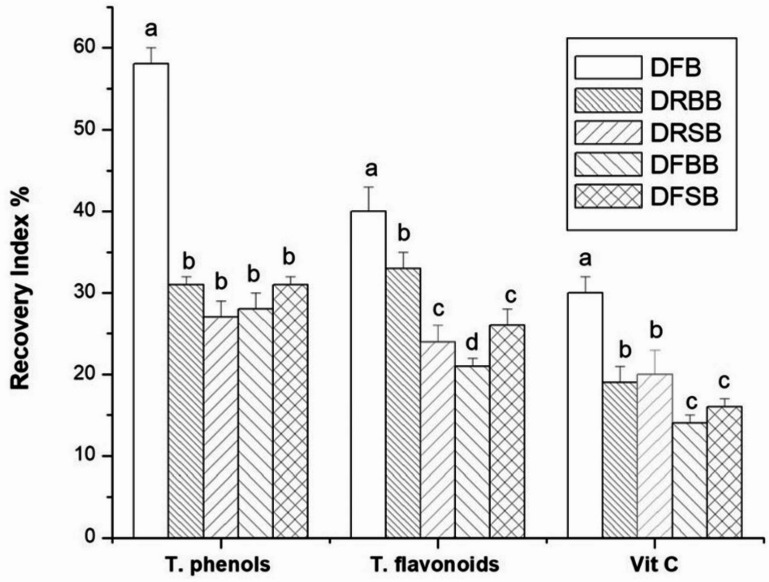
Recovery indices (%) of total phenols, flavonoids, and vitamin C after simulated gastrointestinal digestion of broccoli. *Values are presented as the means (*n* = 3) ± SDs. Values with the same subscribed letters in the same group indicated nonsignificant differences (*p* < 0.05): *DFB* digested fresh broccoli, *DRBB* digested refrigerated boiled broccoli, *DFBB* digested frozen boiled broccoli, *DRSB* digested refrigerated steamed broccoli, *DFSB* digested frozen steamed broccoli.

The effect of* in vitro* gastrointestinal digestion on the bioactive components in broccoli was investigated by calculating the recovery index. As shown in Fig. 2, it was possible to recover 58%, 40%, and 30% of the phenols, flavonoids, and vitamin C, respectively, after *in vitro* gastrointestinal digestion of fresh broccoli. In the *in vitro* gastrointestinal digestion of heat-treated broccoli, there was a significant decrease in the recovery indices of phenols, flavonoids, and vitamin C compared to those of digested fresh broccoli (DFB). There was no significant difference (*p* > 0.05) between the total phenol content of DRBB, DFBB, DRSB, and DFSB and that of DFB (Fig. 2). Moreover, vitamin C was the most affected bioactive compound, as the lowest recovery index was recorded in the digested frozen samples (DFBB and DFSB), whether treated with boiled water or steam (14% and 16%, respectively). This finding was in agreement with those mentioned by Scrob et al.^5^ and Vallejo et al.^38^. They demonstrated that vitamin C was the metabolite that showed a greater decrease (91% loss) after simulated *in vitro* gastrointestinal digestion of broccoli. Their study revealed that while flavonoids and phenolics were relatively stable during gastric digestion, they underwent significant degradation during the intestinal phase. Vitamin C, being highly sensitive to oxidative and alkaline conditions, was particularly vulnerable under simulated intestinal environments.

### Antioxidant capacity of bioactive components

Table 2 illustrates the impact of different cooking methods (boiling and steaming) and storage conditions (refrigerated and frozen) on the antioxidant capacity, including the antiradical activity (ARA%), ferric-reducing antioxidant power (FRAP %) and retained activity (RA%) of broccoli. A significant reduction (*p* ≤ 0.05) in refrigerated boiled broccoli (RBB) was recorded compared to that in fresh broccoli. In RBB, the ARA percentage significantly decreased from 67.4% in fresh broccoli to 37.9%, and the FRAP % dropped from 130 to 80%. Moreover, for refrigerated boiled broccoli, the RA was 56.2% for ARA and 60.6% for FRAP. For frozen boiled broccoli (FBB), the ARA percentage decreased significantly to 29.1%, and the FRAP decreased to 52.5%, with retained activities of 43.2% for ARA and 39.8% for FRAP. Steaming broccoli resulted in a significant decrease in ARA to 37.1% and FRAP to 81.7% in refrigerated samples. The retained activities were 55.0% for DPPH and 61.9% for FRAP. In the case of frozen steamed broccoli (FSB), the ARA decreased to 30.4% and the FRAP decreased to 47.5%, with retained activities of 45.1% for ARA and 35.9% for FRAP.

**Table 2:** The combined effect of heat treatment and cold storage on the antioxidant potential of ready-to-eat broccoli and retained activity.

Treatment	DPPH* ARA (%)	%RA	FRAP* %	%RA
Fresh broccoli (FB)	67.4 ± 0.03^a^	–	132 ± 0.03^a^	–
Refrigerated broccoli				
Boiled (RBB)	37.9 ± 0.02^b^	56.2	80.0 ± 0.03^b^	60.6
Steamed (RSB)	37.1 ± 0.04^b^	55.0	81.7 ± 0.03^b^	61.9
Frozen broccoli				
Boiled (FBB)	29.1 ± 0.0^c^	43.2	52.5 ± 0.02^c^	39.8
Steamed (FSB)	30.4 ± 0.09^c^	45.1	47.5 ± 0.01^cd^	35.9

*FRAP* ferric-reducing antioxidant power, *DPPH* 2,2-diphenyl-2-picryl-hydrazyl, *RA* retained activity, *ARA* antiradical activity.

*Values are presented as the means (*n* = 3) ± SDs. Values with the same subscribed letters in the same column indicate nonsignificant differences (*p* < 0.05).

Boiling resulted in a significant reduction in antioxidant capacity, likely due to the leaching of active compounds into the cooking water. According to^39^, boiling causes a considerable loss of bioactive compounds due to their leaching into cooking water. Although steaming also led to a reduction in antioxidant capacity, the loss was less than that resulting from boiling. Steaming better preserves active compounds because food does not directly contact water, reducing the leaching of beneficial compounds. In general, the retained activities ranged between 45.1 and 55.0% for ARA and 35.9% and 61.9% for FRAP. This was in agreement with Miglio et al.^40^, who noted that steaming is one of the best cooking methods for preserving the nutritional content of vegetables. Additionally, Renard et al.^39^ reported that heat treatment with steam conserves bioactive substances more than boiling does.

It is known that the effects of freezing on phytochemicals are multifactorial, encompassing both losses and potential increases depending on the nature of the compound and its localization within the plant tissue. Freezing can disrupt cellular structures through ice crystal formation, which may lead to the degradation of sensitive compounds such as vitamin C and certain flavonoids. However, this disruption may also enhance the extractability of bound phenolics by breaking down cell walls, increasing their bioaccessibility^11^. In the present study, compared with refrigerated storage, frozen storage resulted in a greater reduction in antioxidant capacity, regardless of whether the broccoli was boiled or steamed. This may be due to the impact of freezing and thawing on the cellular structure of broccoli, making it more susceptible to the loss of active compounds. In general, freezing can cause cellular damage in plant tissues, leading to nutrient loss^39^. Our results indicate that under the tested conditions, the degradative effects of freezing outweighed any potential gains, as evidenced by the significant reduction in antioxidant activity and phenolic content in frozen samples.

**Fig. 3:**
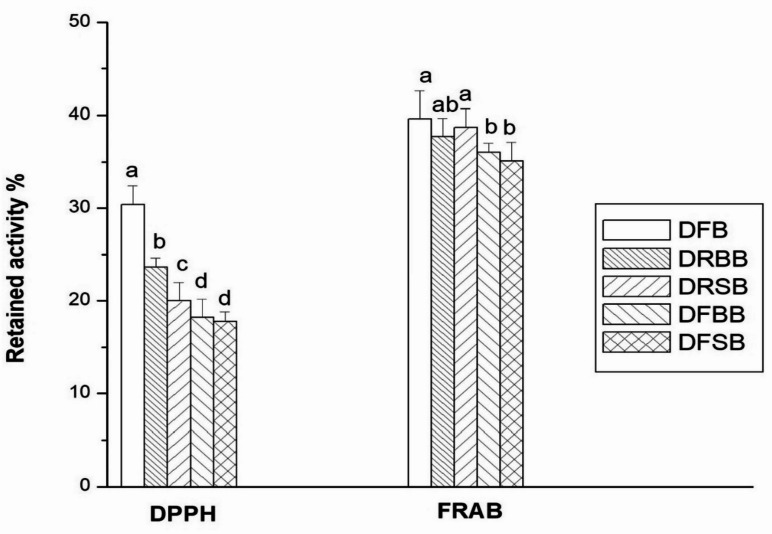
The combined effect of heat treatment and cold storage on the retained antioxidant activity of ready-to-eat broccoli after simulated gastrointestinal digestion of broccoli. *Values are presented as the means (*n = 3*) ± SDs. Values with the same subscribed letters in the same group are not significantly different (*p* < 0.05). *FRAP* ferric-reducing antioxidant power, *DPPH* 2,2-diphenyl-2-picryl-hydrazyl, *RA* retained activity. *DFB* digested fresh broccoli, *DRBB* digested refrigerated boiled broccoli, *DFBB* digested frozen boiled broccoli, *DRSB* digested refrigerated steamed broccoli, *DFSB* digested frozen steamed broccoli.

Figure 3 showed the combined effect of heat treatment and cold storage on the retained antioxidant activity of ready-to-eat broccoli after simulated gastrointestinal digestion of broccoli. In the DPPH assay, DFB had the highest retained antioxidant activity at 30%, indicating it is significantly different from all other treatments (*p* < 0.05). DRBB showed 24%, then DRSB with 19.5%, showing a gradual decline. The lowest values were observed in DFBB and DFSB, both around 18%, meaning they are statistically different. These results clearly show that freezing when combined with boiling or steaming, leads to a significant reduction in radical scavenging activity compared to fresh or refrigerated samples.

The retained antioxidant activity measured by the FRAP assay was highest in DFB and DRSB, showing 39 and 39.2% retained activity, respectively, and were not significantly different from each other. DRBB, DFBB, and DFSB retained around 37% of their activity, (*p < 0.05*). This result suggests that fresh broccoli and refrigerated steamed broccoli are the most effective in preserving antioxidant power after digestion, while boiling and freezing treatments still maintain good levels but with significantly lower retention.

The observed reduction in antioxidant capacity and phenolic content after *in vitro *digestion is not solely due to compound degradation, but also reflects complex biochemical interactions^41^. Phenolic compounds exist in free, conjugated, and matrix-bound forms, and their bioaccessibility is influenced by the extent of release during digestion. Interactions with digestive enzymes may lead to structural transformation or inactivation of certain phenolics. Additionally, *phenolics can bind to proteins and polysaccharides* within the food matrix, forming insoluble complexes or becoming physically entrapped, which limits their accessibility and antioxidant function. These interactions can be synergistic or antagonistic, depending on the compound structure and matrix composition. Such mechanisms help explain the variability in antioxidant retention and phenolic recovery across different broccoli treatments and digestion stages. These findings align with literature emphasizing the superior antioxidant properties of fresh vegetables. A recent broad analysis found that minimally processed plant-based foods maintain significantly higher antioxidant capacity than processed or ultra-processed counterparts. Moreover, a targeted study comparing fresh and fresh-cut vegetables showed that fresh samples generally contained higher phenolic acid levels, underscoring how even mild processing can compromise phytochemical content^42,43^.

### Dietary fibers of broccoli samples before and after *in vitro* gastrointestinal digestion

Table 3 presents the dietary fiber content in different broccoli samples subjected to various heat treatments and storage conditions. The dietary fibers are categorized into total dietary fiber (TDF), soluble dietary fiber (SDF), and insoluble dietary fiber (IDF). Fresh broccoli (FB) had the highest TDF (2.86%), which was significantly (*p* ≤ 0.05) greater than that of FSB (2.28%). Other treatments (RBB, RSB, FBB) had TDF values ranging between 2.58 and 2.62%, with no significant differences from those of FB but significantly greater values than those of FSB. FB had the highest SDF content, at 1.84%. Frozen broccoli (FBB and FSB) had lower SDF values (1.48 and 1.23%, respectively), which were significantly lower than those of FB. Refrigerated broccoli (RBB and RSB) has SDF values of 1.66 and 1.60%, respectively, which are not significantly different from those of FB but are greater than those of frozen samples.

**Table 3:** Effect of heat treatments and storage on dietary fibers in broccoli.

Treatment	% Dietary fibers*		
	Total (TDF)	Soluble (SDF)	Insoluble (IDF)
Fresh broccoli (FB)	2.86 ± 0.15^a^	1.84 ± 0.09^a^	1.02 ± 0.11^a^
Refrigerated broccoli			
Boiled (RBB)	2.62 ± 0.14^a^	1.166 ± 0.14^ab^	0.94 ± 0.06^b^
Steamed (RSB)	2.59 ± 0.03^a^	1.60 ± 0.08^ab^	0.99 ± 0.06^b^
Frozen broccoli			
Boiled (FBB)	2.58 ± 0.14^a^	1.48 ± 0.1^b^	1.1 ± 0.09^a^
Steamed (FSB)	2.28 ± 0.16^b^	1.23 ± 0.04^c^	1.05 ± 0.08^a^

*FB* fresh broccoli, *RBB* refrigerated boiled broccoli, *FBB* frozen boiled broccoli, *RSB* refrigerated steamed broccoli, *FSB* frozen steamed broccoli.

*Values are presented as the means (*n* = 3) ± SDs. Values with the same subscribed letters in the same group are not significantly different (*p* < 0.05).

FB had the highest IDF content, at 1.02% ± 0.11, which was not significantly different from those of FBB (1.1% ± 0.09) and FSB (1.05% ± 0.08). RBB and RSB had lower IDF values (0.94% ± 0.06 and 0.99% ± 0.06, respectively), both of which were significantly different from those of FB.

The results indicate that fresh broccoli generally retains the highest levels of dietary fibers (TDF, SDF, and IDF) compared to other forms subjected to heat treatments and storage. The reduction in dietary fiber content, particularly soluble fiber, is more noticeable in frozen and steamed broccoli. This suggests that the combination of freezing and steaming may degrade the fiber content more than other treatments. These findings are consistent with previous studies that demonstrated the impact of processing and storage on the nutritional quality of vegetables. These findings underscore the importance of considering not only the quantitative changes in dietary fiber but also their physiological implications. Building on this, Wachtel-Galor et al.^44^ emphasized that cooking and storage could significantly alter the fiber content in vegetables, where the fresh forms retain the highest nutritional value. Similarly, Zhang et al.^45^ reported that steaming and boiling could lead to a reduction in both soluble and insoluble fiber contents in broccoli. Beyond composition, it is important to note that dietary fibers serve as key substrates for gut microbiota. Fibers of varying solubility exert distinct effects on microbial community structure and colonic barrier integrity, thereby influencing host health outcomes^46^. The observed increase in insoluble dietary fiber (IDF) following boiling, even exceeding the values found in fresh broccoli, may be attributed to several factors. One explanation is the thermal transformation of soluble fiber into insoluble fractions, as heat can restructure polysaccharides and promote aggregation or cross-linking, making them less soluble. This phenomenon has been documented in cruciferous vegetables, including broccoli, where cooking alters the fiber matrix and shifts the balance between soluble and insoluble fractions^47^. Additionally, cooking may lead to the formation of resistant compounds, such as thermally modified lignin-like structures or Maillard reaction products, which are quantified as part of the insoluble fiber fraction^48^. Analytical artifacts, including incomplete solubilization or precipitation during sample preparation, may also contribute to this trend. In general, thermal processing and storage significantly influence the structural integrity and composition of dietary fiber in vegetables such as broccoli^47,49^. Heat treatments, including boiling and steaming, can induce partial solubilization of insoluble dietary fibers and degrade polysaccharides through hydrolysis and depolymerization^50^, leading to a shift in the balance between soluble and insoluble fractions. These changes are often attributed to the breakdown of cell wall components, including pectins, hemicelluloses, and cellulose, which are sensitive to temperature and moisture conditions. Additionally, freezing disrupts cellular structures via ice crystal formation, which can further compromise fiber integrity and reduce extractability^49^. Structural modifications such as the loss of arabinans and galactans from pectic side chains, and the incorporation of syringyl units into lignin polymers, have been observed during storage and thermal treatment, contributing to altered fiber profiles and reduced nutritional functionality^50^. These mechanisms collectively explain the observed reductions in soluble dietary fiber and shifts in total fiber content across different broccoli treatments.

Table 4 presents the dietary fiber content of various broccoli samples after *in vitro* gastrointestinal digestion. The digested fresh broccoli (DFB) had the highest TDF content, at 2.89%. The DFB treatment had the lowest TDF content, at 2.58%, which was significantly lower than that of DFB. The TDF values for DRBB, DRSB, and DFBB ranged from 2.8 to 2.81%, which are lower than those of DFB but higher than those of DFSB.

**Table 4:** Effect of *in vitro* gastrointestinal digestion on dietary fibers in broccoli.

Treatment	%Dietary fibers*		
	Total (TDF)	Soluble (SDF)	Insoluble (IDF)
Fresh broccoli (FB)	2.89 ± 0.05^a^	1.59 ± 0.04^a^	1.3 ± 0.02^b^
Refrigerated broccoli			
Boiled (RBB)	2.81 ± 0.04^b^	1.4 ± 0.04^b^	1.41 ± 0.06^a^
Steamed (RSB)	2.81 ± 0.03^b^	1.46 ± 0.08^b^	1.35 ± 0.06^b^
Frozen broccoli			
Boiled (FBB)	2.8 ± 0.04^b^	1.38 ± 0.05^c^	1.42 ± 0.03^a^
Steamed (FSB)	2.58 ± 0.06^c^	1.23 ± 0.06^d^	1.35 ± 0.02^b^

*FB* fresh broccoli, *RBB* refrigerated boiled broccoli, *FBB* frozen boiled broccoli, *RSB* refrigerated steamed broccoli, *FSB* frozen steamed broccoli.

*Values are presented as the means (*n* = 3) ± SDs. Values with the same subscribed letters in the same group are not significantly different (*p* < 0.05).

DFB had the highest SDF content, at 1.59%. DFBB and DFSB had lower SDF values (1.38% and 1.23%, respectively), which were significantly different from those of DFB. DRSB had an SDF value of 1.46% ± 0.08, which was also lower than that of DFB but greater than that of DFBB. DFSB had the lowest SDF content, at 1.23% ± 0.06, which was significantly lower than that of DFB and DFBB. DRBB had the highest IDF content at 1.41% ± 0.06. DFB had an IDF of 1.3% ± 0.02, which was significantly lower than that of DRBB but similar to that of DRSB and DFSB. The IDF values for DRSB and DFSB are not significantly different from each other or from those for DFB. The IDF content of DFBB was 1.42% ± 0.03, similar to that of DRBB.

The data in Table 4 indicate that fresh broccoli retains the highest levels of total and soluble dietary fibers after *in vitro* gastrointestinal digestion, highlighting its nutritional robustness. However, the cooking methods (boiling and steaming) and storage conditions (refrigeration and freezing) appear to significantly impact the dietary fiber content. Notably, frozen steamed broccoli (DFSB) had the lowest dietary fiber content across all the samples.

These results are consistent with previous research on the impact of food processing on fiber retention. Studies by Wachtel-Galor et al.^44^ and Zhang et al.^45^ have documented similar trends, where fresh vegetables typically retain higher nutritional quality postdigestion than their processed counterparts, regarding the effects of gastrointestinal digestion, the process can modify fiber fractions through solubilization, partial degradation of polysaccharides, and the generation of resistant residues. These transformations shift the balance between soluble and insoluble fibers, as seen in the increase in IDF and decrease in SDF post-digestion. A study on broccoli stems confirmed that digestion alters oligosaccharide composition, influencing fiber functionality^51^. However, measuring fiber fractions alone does not fully capture nutritional robustness. A comprehensive assessment should consider not only compositional changes but also the physiological functionality of fiber^52,53^.

### HPLC analysis of phenolic compounds

The bioactive components before and after *in vitro* gastrointestinal digestion of boiled broccoli stored under refrigeration or freezing conditions are presented in Table 5. The fresh broccoli used in the current study is characterized by its high content of vanillic acid, pyrogallol, caffeine, catechol, and P-OH-benzoic acid (Table 5). Compared to fresh broccoli (FB), all heat-treated broccoli generally exhibited a decrease in phenolic content for most bioactive compounds. For instance, in boiled broccoli, the vanillic acid content decreased from 6415.6 μg/100 g in FB to 4440 and 3750.8 μg/100 g in RBB and FBB, respectively (Table 5). On the other hand, in steamed broccoli, the vanillic acid content decreased from 6415.6 μg/100 g in FB to 4604.2 and 3870.2 μg/100 g in RSB and FSB, respectively (Table 6). Similar trends were observed for the other studied bioactive compounds in boiled broccoli (Table 5) and steamed broccoli (Table 6). These results indicate that cooking with boiling water has a stronger negative effect on the phenolic content of broccoli than cooking with steam. This can be interpreted as the boiling process involving the submergence of broccoli in hot water, which can cause the leaching of water-soluble compounds, including phenolic compounds, into cooking water. This loss of phenolic compounds during boiling contributes to the reduction in phenolic content observed in RBB and FBB compared to FB. The reduction in phenolic content during boiling is consistent with the findings of Wu et al.^54^. They mentioned that boiling involves exposure to high temperatures and water, which can lead to the degradation and leaching of phenolic compounds from vegetables such as broccoli. Compared with refrigeration, freezing generally exacerbates the loss of phenolic compounds after boiling or steaming. This may be due to the formation of ice crystals, which can disrupt cell structures and lead to the release of phenolic compounds from the food matrix^10^.

**Table 5:** Bioactive components before and after *in vitro* gastrointestinal digestion of boiled broccoli stored under refrigerated or frozen conditions.

Phenols μg/100 g	Fresh broccoli			Boiled-broccoli					
				Refrigerated			Frozen		
	FB	DFB	%loss	RSB	DRSB	%loss	FSB	DFSB	%loss
Vanillic acid	6415.6	1122.6	82.5	4440.0	448.2	93.0	3750.8	395.5	93.8
Pyrogallol	5738.6	884.6	84.6	4130.2	501.65	91.3	2708.6	395.6	93.1
Caffeine	1275.0	510	60.0	688.7	455.5	64.3	689.2	312.8	75.5
Catechol	1200.2	773.1	35.6	812.27	122.2	89.8	687.7	120.2	90.0
Chlorogenic acid	482.5	122.15	74.7	224.8	88.8	81.6	124.8	64.3	86.7
P-OH- benzoic	1210.1	91.2	92.5	882.5	60.5	95.0	680.5	31.5	97.4
Catechein	450	111.7	75.2	380.4	58.4	87.0	220.4	68.8	84.7
Ferulic acid	99.08	95.23	3.9	93.59	56.01	43.5	96	32.21	67.5
Ellagic acid	84.02	40.55	51.7	54.55	33.2	60.5	35.3	15.9	81.1
Gallic acid	188.2	32.0	83.0	121.3	15.2	91.9	33.9	7.87	95.8
4-Amino-benzoic acid	99.29	38.20	61.5	52.0	13.9	86.0	41.29	9.7	90.2
Coumarin	12.5	3.3	73.6	8.2	3.6	71.2	6.6	0.0	100.0
Average			64.9			79.6			88.0

*%loss* loss percentage after digestion of each counterpart sample, *FB* fresh broccoli, *DFB* digested fresh broccoli, *RBB* refrigerated boiled broccoli, *DRBB* digested refrigerated boiled broccoli, *FBB* frozen boiled broccoli, *DFBB* digested frozen boiled broccoli.

**Table 6:** Bioactive components before and after *in vitro* gastrointestinal digestion of steamed broccoli stored under refrigerated or frozen conditions.

Phenols μg/100 g	Fresh broccoli			Steamed-broccoli							
				Refrigerated				Frozen			
	FB	DFB	%loss	RSB	%loss	DRSB	%loss	FSB	%loss	DFSB	%loss
Vanillic acid	6415.6	1122.6	82.5	4604.2	36.0	582.2	90.9	3870.2	39.7	387.8	94.0
Pyrogallol	5738.6	884.6	84.6	4238.5	26.1	533.6	90.7	2938.5	48.8	388.5	93.2
Caffeine	1275.0	510	60.0	775.5	39.2	421.0	67.0	677.3	46.9	387.6	69.6
Catechol	1200.2	773.1	35.6	822.7	31.5	322.0	73.2	722.7	39.8	128.27	89.3
Chlorogenic acid	482.5	122.2	74.7	324.3	32.8	124.3	74.2	249.8	48.2	84.3	82.5
P-OH-benzoic	1210.1	91.2	92.5	911.7	24.7	81.9	93.2	880.7	27.2	67.7	94.4
Catechein	450	111.7	75.2	320.3	28.8	77.4	82.8	370.0	17.8	71.7	84.1
Ferulic acid	99.08	95.2	3.9	98.1	1.0	44.2	55.4	96.2	2.9	46.2	53.4
Ellagic acid	84.02	40.5	51.7	59.65	29.0	26.63	68.3	42.0	50.0	27.52	67.2
Gallic acid	188.2	32.0	83.0	122.3	35.0	25.8	86.3	112.6	40.2	17.2	90.9
4-Amino-benzoic acid	99.29	38.2	61.5	40.9	58.8	22.9	76.9	39.9	59.8	12.7	87.2
Coumarin	12.5	3.3	73.6	10	20.0	3.5	72.0	7.3	41.6	4.5	64.0
Average			64.9		29.6		77.6		38.6		80.8

*%loss* reduction percentage after each treatment, *FB* fresh broccoli, *DFB* digested fresh broccoli, *RSB* refrigerated steamed broccoli, *DRSB* digested refrigerated steamed broccoli, *FSB* frozen steamed broccoli, *DFSB* digested frozen steamed broccoli.

After *in vitro* gastrointestinal digestion, the phenolic content of fresh broccoli generally decreased compared to that of its undigested counterpart. For instance, the pyrogallol content decreased from 5738.6 μg/100 g in FB to 884.6 μg/100 g in DFB (Table 5). Similarly, after digestion, refrigerated boiled broccoli (RBB) also exhibited a decrease in phenolic content compared to its undigested counterpart. For instance, the pyrogallol content decreased from 4130.2 μg/100 g in RBB to 501.65 μg/100 g in DRBB. Similarly, compared with its undigested counterpart, frozen boiled broccoli (FBB) showed a decrease in phenolic content after digestion. For instance, the pyrogallol content decreased from 2708.6 μg/100 g in FBB to 395.6 μg/100 g in DFBB. Similar trends were observed for the other studied phenolic compounds in boiled broccoli (Table 5).

For steamed broccoli, the pyrogallol content decreased from 4238.5 μg/100 g in the RSB to 533.6 μg/100 g in the DRSB. Moreover, compared with its undigested counterpart, frozen steamed broccoli (FBB) showed a decrease in phenolic content after digestion. For instance, the pyrogallol content decreased from 2938.5 μg/100 g in FSB to 388.5 μg/100 g in DFSB. Similar trends were observed for the other studied phenolic compounds in steamed broccoli (Table 6).

The percentage of phenol loss after digestion of each sample was calculated compared to that of its counterpart and is shown in Tables 5 and 6. The average loss of phenolics in freshly digested broccoli was 64.9%, while it was 79.6% in DRBB and 88% in DFBB (Table 5). For steamed broccoli, the loss percentages in DRSB and DFSB were 77.6 and 80.8%, respectively (Table 6). This finding was in agreement with those mentioned in a previous study, where the percentage loss of bioactive substances, especially phenols, reached 75 to 91% after gastrointestinal digestion of broccoli^5^. Several other previous studies demonstrated a decrease in the concentration and bioaccessibility of phenolic compounds after *in vitro *gastrointestinal digestion of different vegetables and fruits^6,45,55–57^.

Generally, the observed reductions in bioactive compound levels and antioxidant capacity following thermal processing, storage, and *in vitro* digestion have likely been caused by a combination of physicochemical and enzymatic mechanisms. It is probable that leaching during boiling has led to the migration of water-soluble compounds such as vitamin C and phenolics into the cooking medium, thereby reducing their concentration in the vegetable matrix^40^. Oxidation processes, particularly under high-temperature conditions, may have contributed to the degradation of sensitive compounds, especially polyphenols and ascorbic acid, through the generation of reactive oxygen species (ROS)^58^. Additionally, cell wall disruption during cooking and freezing has possibly altered the structural integrity of plant tissues, facilitating the release of intracellular compounds while exposing them to further degradation^37^. During digestion, enzymatic interactions are believed to have played a critical role in transforming complex phenolics into simpler metabolites, which may exhibit different bioactivities or bioaccessibility profiles. These mechanisms have been widely reported in recent literature and are thought to collectively influence the nutritional value of plant-based foods through both processing and digestive transformations^59^. These findings highlight the importance of considering both processing and digestive transformations when evaluating the nutritional value of plant-based foods.

## Conclusion

This study shows that cooking, storage, and digestion significantly affect the nutritional quality of ready-to-eat broccoli. Boiling and freezing caused the greatest losses in vitamin C, phenolics, and antioxidant activity, while steaming and refrigeration helped preserve these compounds. Fresh broccoli consistently had the highest antioxidant capacity and soluble fiber, which are important for gut health. Boiling increased insoluble fiber, likely due to structural changes in polysaccharides or the formation of resistant compounds. Digestion further shifted fiber composition, reducing soluble and increasing insoluble fractions. These changes reflect how processing and digestion interact with the food matrix and compound structure.

Overall, the findings highlight the importance of gentle cooking and proper storage to maintain the nutritional and functional value of broccoli, with implications for dietary choices and product development. Moreover, simulated gastrointestinal digestion plays a crucial role in revealing the true stability and availability of bioactive compounds. It allows for the assessment of how compounds behave under physiological conditions, offering insights into their potential effectiveness after consumption.

## Supplementary Information

Below is the link to the electronic supplementary material.

Supplementary Material 1

## Data Availability

All data generated or analyzed during this study are included in this published article and its supplementary information (S1).
