# Supplementary material for: Evaluation of bioaccessibility of bioactive compounds in ready-to-eat refrigerated and frozen broccoli using in vitro digestion models
Source: Sci Rep. 2025 Nov 21;15:41457. doi: 10.1038/s41598-025-26034-9 (PMC12645050; doi:10.1038/s41598-025-26034-9)
Supplement: Supplementary file 1 — Supplementary Material 1 [file 41598_2025_26034_MOESM1_ESM.pdf]

Total Phenols content (TPC) calculation

Standard Curve Gallic

Concentration (µl/ml)	Absorbance (OD765)
50	0.156
100	0.301
150	0.589
250	0.892
400	1.185
550	1.601
650	1.888

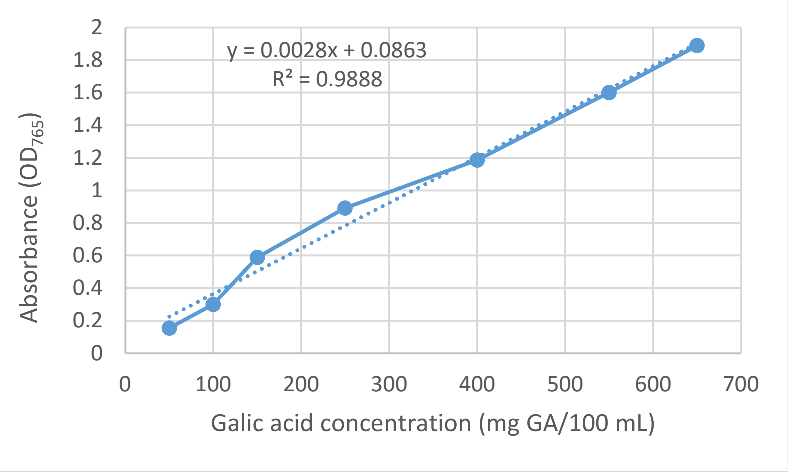

Sample	R (OD)		Mean	StDev	
FB	1.8223	620	610	19.1	A
FB	1.8279	622			
FB	1.7327	588			
RBB	1.6011	541	515	23.9	B
RBB	1.4695	494			
RBB	1.5143	510			
RSB	1.5143	510	503	6.24	B
RSB	1.4807	498			
RSB	1.4891	501			
FBB	1.1475	379	368	11	C
FBB	1.0859	357			
FBB	1.1167	368			
FSB	1.1615	384	393	8.54	C
FSB	1.1895	394			
FSB	1.2091	401			

Statistical analysis

One-way ANOVA: **TPC** versus Sample

Method

Null hypot All means are equal

Alternative Not all means are equal

Significanc α = 0.05

Equal variances were assumed for the analysis.

Factor Information

Factor	Levels	Values
Sample	5	FB, FBB, FSB, RBB, RSB

Analysis of Variance

Source	DF	Adj SS	Adj MS	F-Value	P-Value
Sample	4	116228	29057.1	124.39	0
Error	10	2336	233.6		
Total	14	118564			

Model Summary

S	R-sq	R-sq(adj)	R-sq(pred)
15.284	98.03%	97.24%	95.57%

Means

Sample	N	Mean	StDev	95% CI
FB	3	610	19.1	(590.3, 629.7)
FBB	3	368	11	(348.34, 387.66)
FSB	3	393	8.54	(373.34, 412.66)
RBB	3	515	23.9	(495.3, 534.7)
RSB	3	503	6.24	(483.34, 522.66)

Pooled StDev = 15.2840

Tukey Pairwise Comparisons

Grouping Information Using the Tukey Method and 95% Confidence

Sample	N	Mean	Grouping		
FB	3	610	A		
RBB	3	515		B	
RSB	3	503		B	
FSB	3	393			C
FBB	3	368			C

Means that do not share a letter are significantly different.

Tukey Simultaneous 95% CIs

Interval Plot of TPC vs Sample

Standard Curve quercetin

Concentration (mg/100	Absorbance at 510 nm
20	0.2
60	0.35
100	0.6
140	0.85
200	1.4

Total Flavonoid content (TFC) calculation

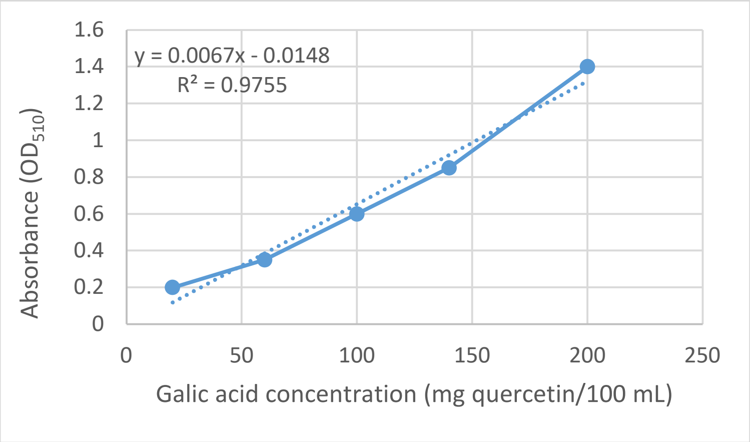

Sample	R (OD)		Mean	StDev	
FB	2.0315	301	295	6	A
FB	1.998	296			
FB	1.9444	288			
RBB	1.194	176	177	5	C
RBB	1.2409	183			
RBB	1.1672	172			
RSB	1.3615	201	198	2	B
RSB	1.3615	201			
RSB	1.3012	192			
FBB	0.9394	138	132	5	D
FBB	0.8858	130			
FBB	0.8724	128			
FSB	0.9528	140	141	5	D
FSB	0.9796	144			
FSB	0.9461	139			

Statistical analysis

One-way ANOVA: **TFC** versus Sample

Method

Null hypot All means are equal

Alternative Not all means are equal

Significanc α = 0.05

Equal variances were assumed for the analysis.

Factor Information

Factor	Levels	Values
Sample	5	FB, FBB, FSB, RBB, RSB

Analysis of Variance

Source	DF	Adj SS	Adj MS	F-Value	P-Value
Sample	4	51039.6	12759.9	469.11	0
Error	10	272	27.2		
Total	14	51311.6			

Model Summary

S	R-sq	R-sq(adj)	R-sq(pred)
5.21536	99.47%	99.26%	98.81%

Means

Sample	N	Mean	StDev	95% CI
FB	3	295	6.56	(288.29, 301.71)
FBB	3	132	5.29	(125.29, 138.71)
FSB	3	141	2.65	(134.29, 147.71)
RBB	3	177	5.57	(170.29, 183.71)
RSB	3	198	5.2	(191.29, 204.71)

Pooled StDev = 5.21536

Tukey Pairwise Comparisons

Grouping Information Using the Tukey Method and 95% Confidence

Sample	N	Mean	Grouping			
FB	3	295	A			
RSB	3	198		B		
RBB	3	177			C	
FSB	3	141				D
FBB	3	132				D

Means that do not share a letter are significantly different.

Tukey Simultaneous 95% CIs

Interval Plot of TFC vs Sample

DFB

Phenols µg/100 g	RT (min)	Area	Amount (µg/100 mL)
**Gallic acid**	2.9	13.4	32
**Catechol**	4.1	62.6	773.1
**Pyrogallol**	4.3	70.0	884.6
**P-OH- benzoic**	4.8	17.3	91.2
**Caffeine**	5.1	45.1	510
**Vanillic acid**	6.2	85.8	1122.6
**4-Amino-benzoic acid**	6.5	13.8	38.2
**Catechein**	7.4	18.7	111.7
**Ferulic acid**	9.27	17.6	95.2
**Chlorogenic acid**	15.1	19.4	122.2
**Ellagic acid**	19.2	13.9	40.5
**Coumarin**	22.5	11.5	3.3

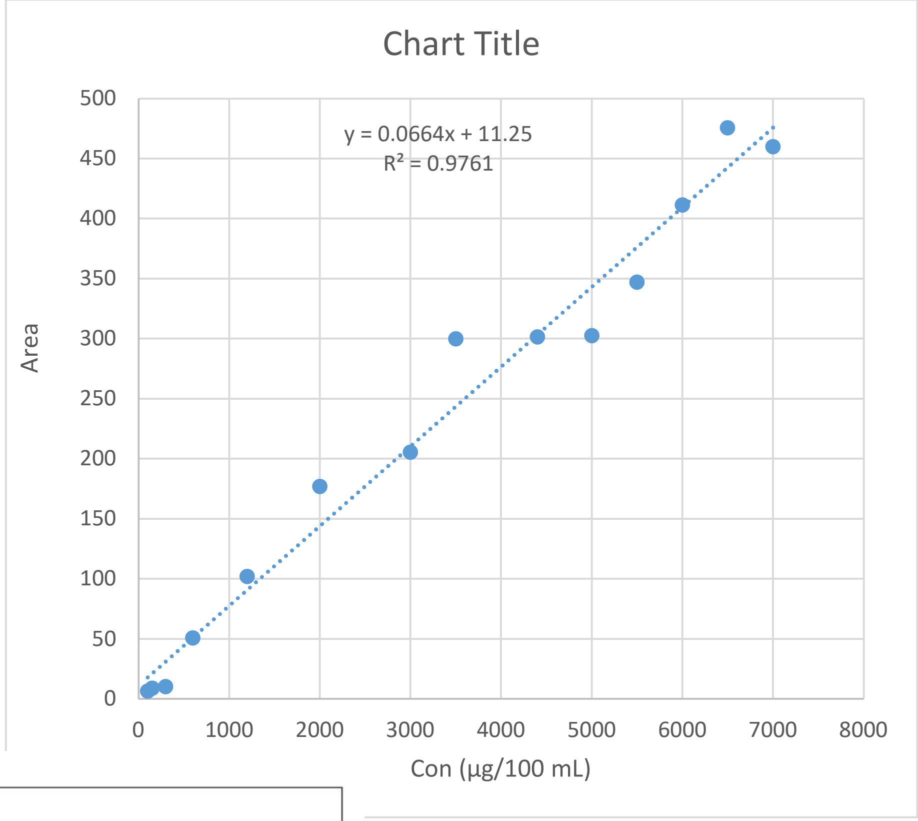

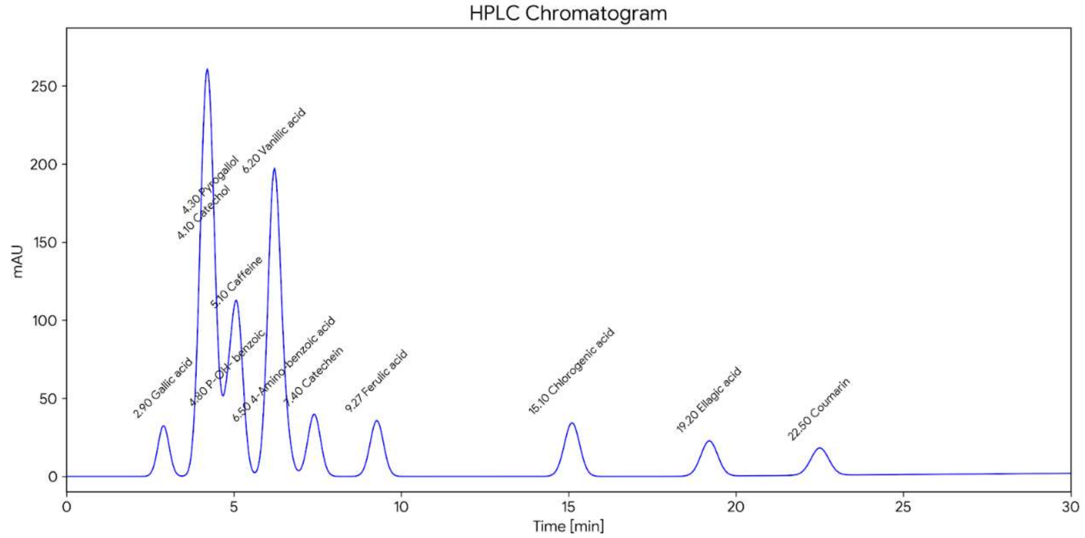

**Standard**		**Experiment**		
Con (µg/10(	Area	**Area**	**Amount (µg/100 mL)**	
100	6.6033	85.8	1122.6	Vanillic acid
150	9.1728	70.0	884.6	Pyrogallol
300	10.4613	45.1	510	Caffeine
600	51.0383	62.6	773.1	Catechol
1200	102.1923	19.4	122.2	Chlorogenic acid
2000	177.0643	17.3	91.2	P-OH-benzoic
3000	205.6543	18.7	111.7	Catechein
3500	299.9493	17.6	95.2	Ferulic acid
4400	301.6803	13.9	40.5	Ellagic acid
5000	302.8343	13.4	32	Gallic acid
5500	347.1293	13.8	38.2	4-Amino-benzoic acid
6000	411.4243	11.5	3.3	Coumarin
6500	475.7193			
7000	460.0143			

RSB

Phenols µg/100 g	RT (min)	Area	Amount (µg/100 mL)
Gallic acid	2.9	19.4	122.3
Catechol	4.1	65.9	822.7
Pyrogallol	4.3	292.7	4238.5
P-OH- benzoic	4.8	71.8	911.7
Caffeine	5.1	62.7	775.5
Vanillic acid	6.2	317.0	4604.2
4-Amino-benzoic acid	6.5	14.0	40.9
Catechein	7.4	32.5	320.3
Ferulic acid	9.27	17.8	98.1
Chlorogenic acid	15.1	32.8	324.3
Ellagic acid	19.2	15.2	59.65
Coumarin	22.5	11.9	10

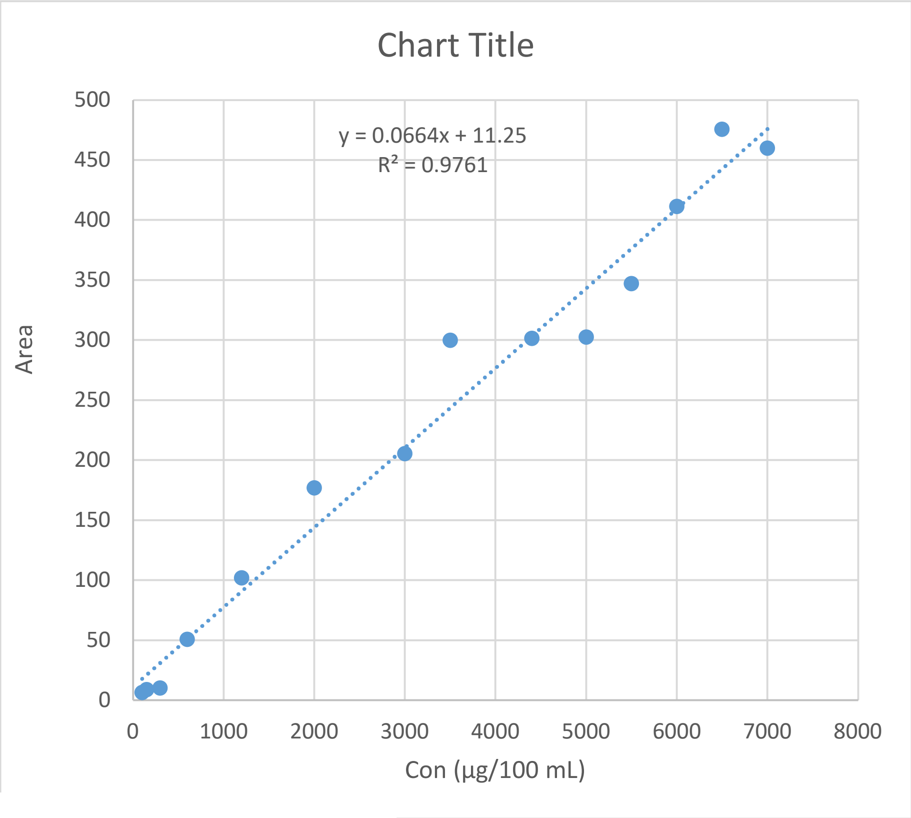

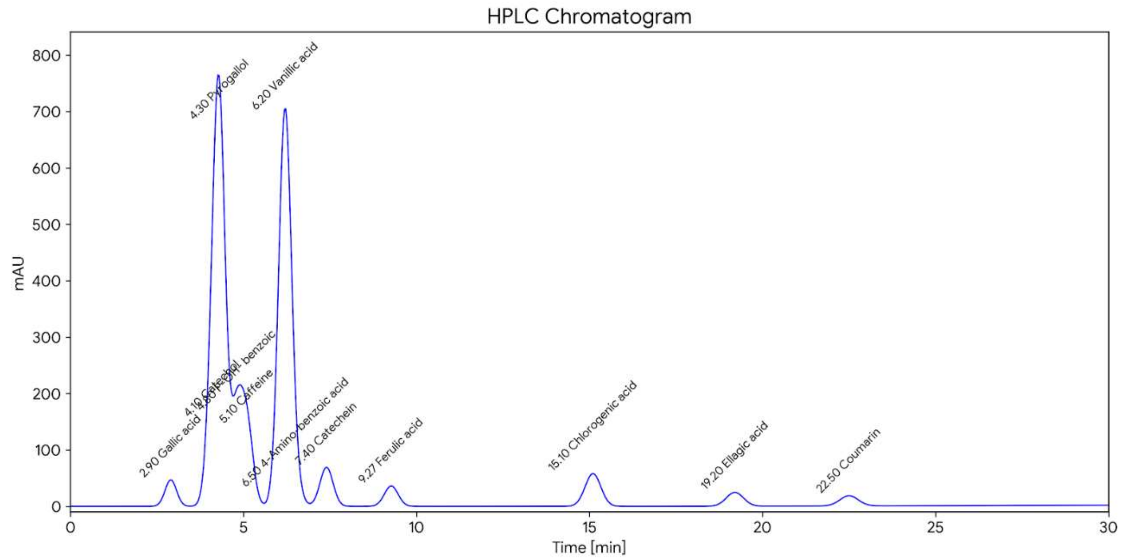

**Standard**		**Experiment**		
Con (µg/10(	Area	**Area**	**Amount (µg/100 mL)**	
100	6.6033	317.0	4604.2	Vanillic acid
150	9.1728	292.7	4238.5	Pyrogallol
300	10.4613	62.7	775.5	Caffeine
600	51.0383	65.9	822.7	Catechol
1200	102.1923	32.8	324.3	Chlorogenic acid
2000	177.0643	71.8	911.7	P-OH-benzoic
3000	205.6543	32.5	320.3	Catechein
3500	299.9493	17.8	98.1	Ferulic
4400	301.6803	15.2	59.65	Ellagic
5000	302.8343	19.4	122.3	Gallic acid
5500	347.1293	14.0	40.9	4-Amino-benzoic acid
6000	411.4243	11.9	10	Coumarin
6500	475.7193			
7000	460.0143			

DRSB

Phenols µg/100 g	RT (min)	Area	Amount (µg/100 mL)
**Gallic acid**	2.9	13.0	25.8
**Catechol**	4.1	32.6	322
**Pyrogallol**	4.3	46.7	533.6
**P-OH- benzoic**	4.8	16.7	81.9
**Caffeine**	5.1	39.2	421
**Vanillic acid**	6.2	49.9	582.2
**4-Amino-benzoic acid**	6.5	12.8	22.9
**Catechein**	7.4	16.4	77.4
**Ferulic acid**	9.27	14.2	44.2
**Chlorogenic acid**	15.1	19.5	124.3
**Ellagic acid**	19.2	13.0	26.63
**Coumarin**	22.5	11.5	3.5

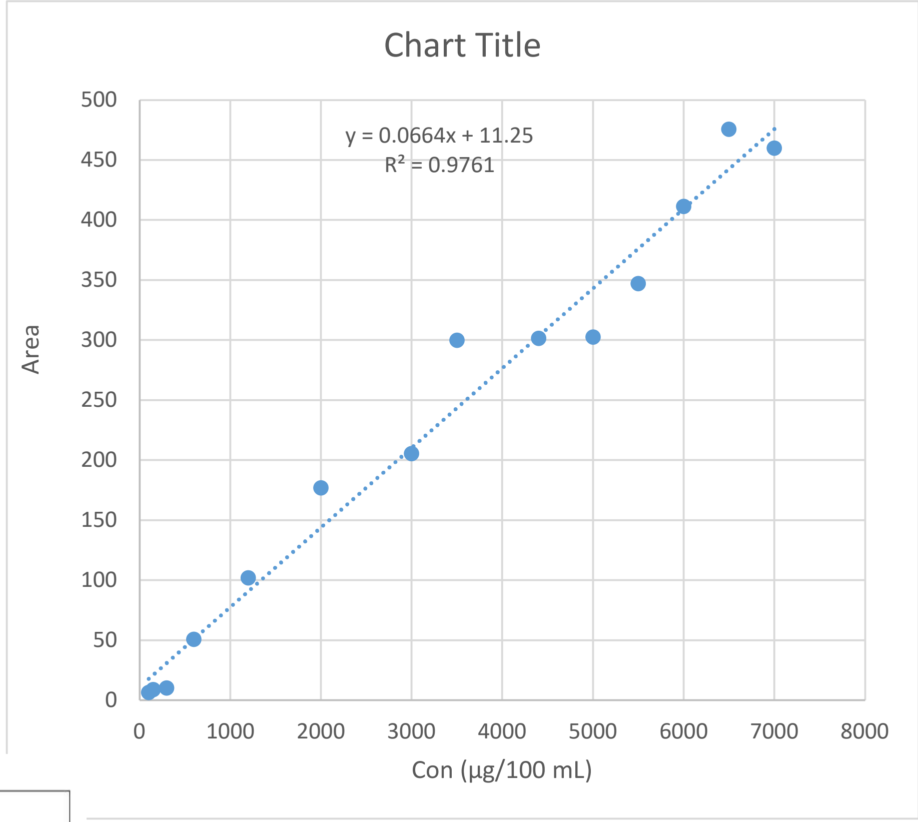

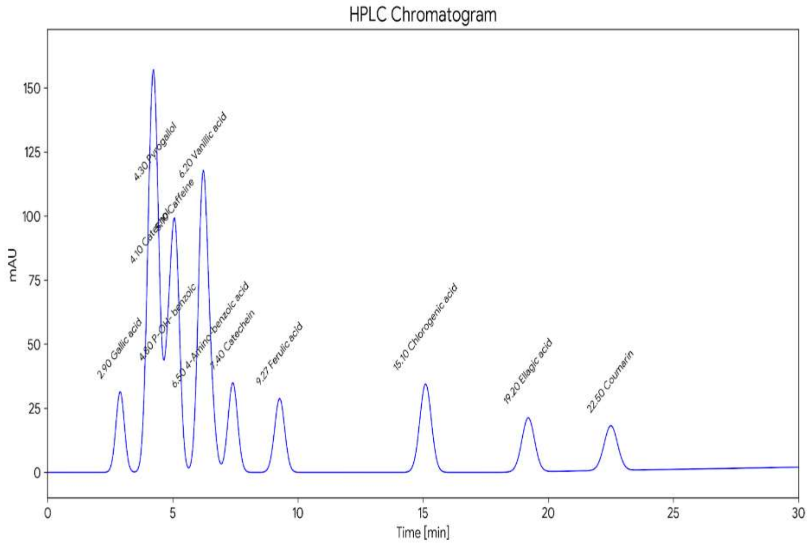

**Standard**		**Experiment**		
Con (µg/10(	Area	**Area**	**Amount (µg/100 mL)**	
100	6.6033	49.9	582.2	Vanillic acid
150	9.1728	46.7	533.6	Pyrogallol
300	10.4613	39.2	421	Caffeine
600	51.0383	32.6	322	Catechol
1200	102.1923	19.5	124.3	Chlorogenic acid
2000	177.0643	16.7	81.9	P-OH-benzoic
3000	205.6543	16.4	77.4	Catechein
3500	299.9493	14.2	44.2	Ferulic acid
4400	301.6803	13.0	26.63	Ellagic acid
5000	302.8343	13.0	25.8	Gallic acid
5500	347.1293	12.8	22.9	4-Amino-benzoic acid
6000	411.4243	11.5	3.5	Coumarin
6500	475.7193			
7000	460.0143			

DRSB

Phenols µg/100 g	RT (min)	Area	Amount (µg/100 mL)
Gallic acid	2.9	13.0	25.8
Catechol	4.1	32.6	322
Pyrogallol	4.3	46.7	533.6
P-OH- benzoic	4.8	16.7	81.9
Caffeine	5.1	39.2	421
Vanillic acid	6.2	49.9	582.2
4-Amino-benzoic acid	6.5	12.8	22.9
Catechein	7.4	16.4	77.4
Ferulic acid	9.27	14.2	44.2
Chlorogenic acid	15.1	19.5	124.3
Ellagic acid	19.2	13.0	26.63
Coumarin	22.5	11.5	3.5

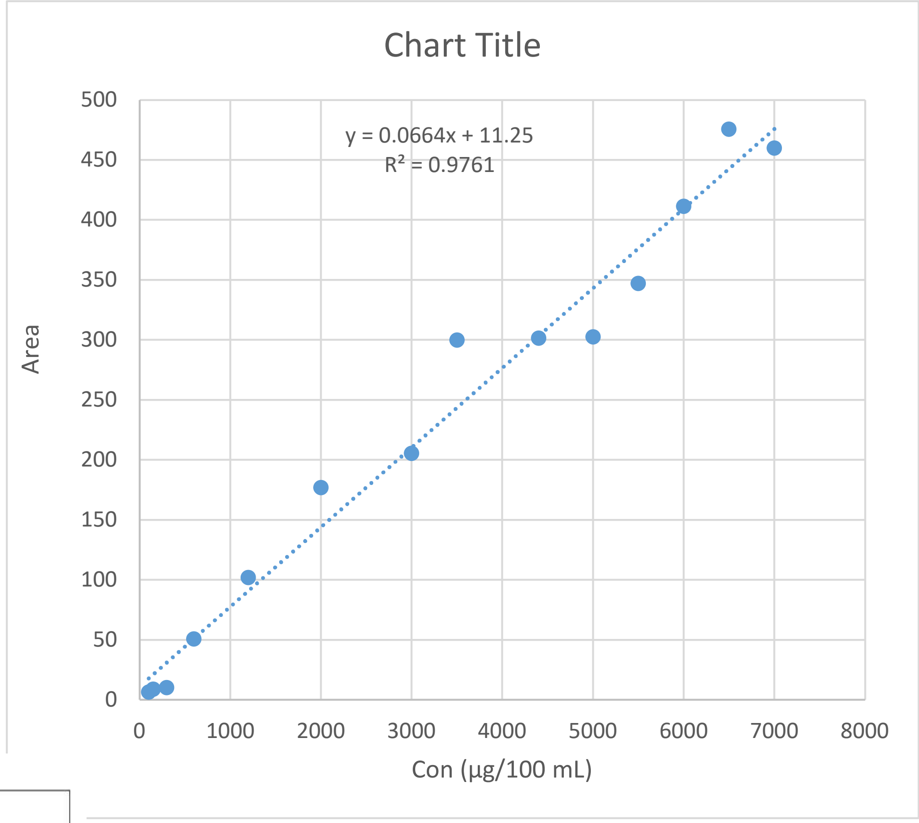

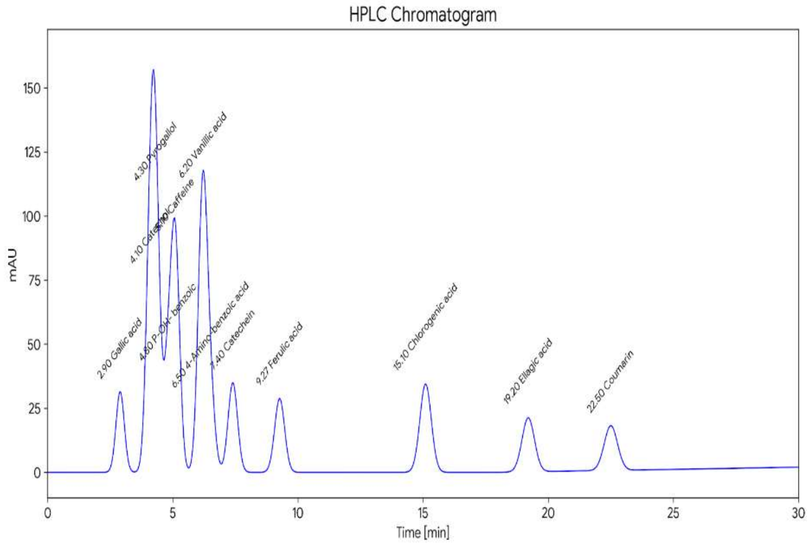

Standard		Experiment		
Con (µg/10(	Area	Area	Amount (µg/100 mL)	
100	6.6033	49.9	582.2	Vanillic acid
150	9.1728	46.7	533.6	Pyrogallol
300	10.4613	39.2	421	Caffeine
600	51.0383	32.6	322	Catechol
1200	102.1923	19.5	124.3	Chlorogenic acid
2000	177.0643	16.7	81.9	P-OH-benzoic
3000	205.6543	16.4	77.4	Catechein
3500	299.9493	14.2	44.2	Ferulic acid
4400	301.6803	13.0	26.63	Ellagic acid
5000	302.8343	13.0	25.8	Gallic acid
5500	347.1293	12.8	22.9	4-Amino-benzoic acid
6000	411.4243	11.5	3.5	Coumarin
6500	475.7193			
7000	460.0143			

FSB

Phenols µg/100 g	RT (min)	Area	Amount (µg/100 mL)
Gallic acid	2.9	18.7	112.6
Catechol	4.1	59.2	722.7
Pyrogallol	4.3	206.4	2938.5
P-OH- benzoic	4.8	69.7	880.7
Caffeine	5.1	56.2	677.3
Vanillic acid	6.2	268.2	3870.2
4-Amino-benzoic acid	6.5	13.9	39.9
Catechein	7.4	35.8	370
Ferulic acid	9.27	17.6	96.2
Chlorogenic acid	15.1	27.8	249.8
Ellagic acid	19.2	14.0	42
Coumarin	22.5	11.7	7.3

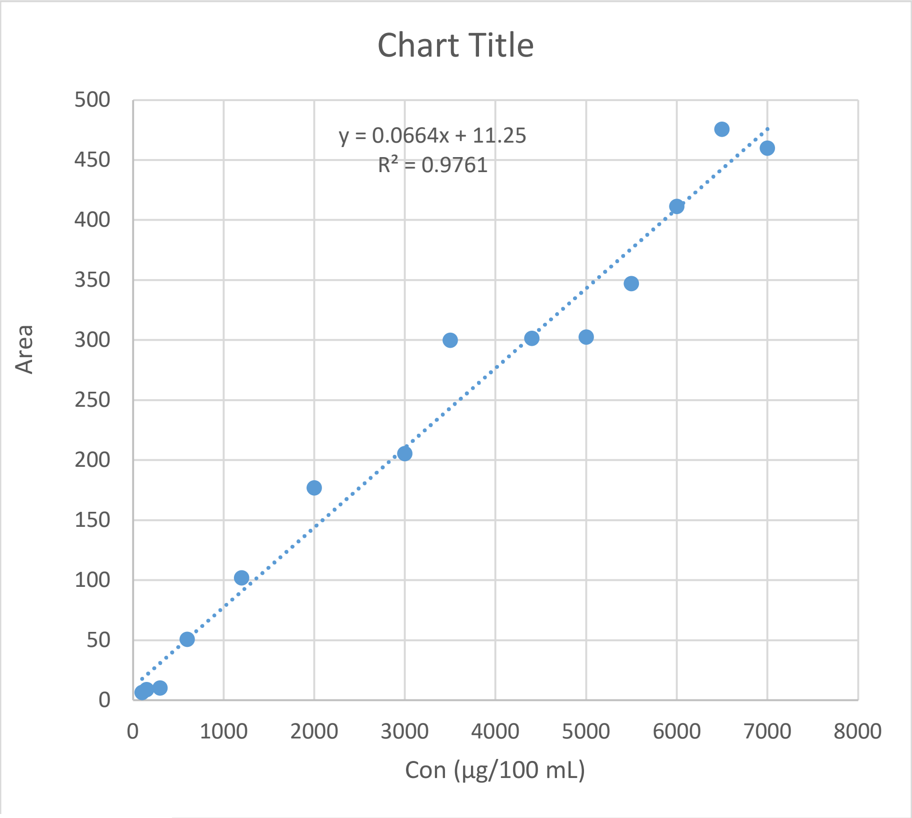

Standard **Experiment**

Con (µg/10(	Area	Area	Amount (µg/100 mL)	
100	6.6033	268.2	3870.2	Vanillic acid
150	9.1728	206.4	2938.5	Pyrogallol
300	10.4613	56.2	677.3	Caffeine
600	51.0383	59.2	722.7	Catechol
1200	102.1923	27.8	249.8	Chlorogeni c acid
2000	177.0643	69.7	880.7	P-OH- benzoic
3000	205.6543	35.8	370	Catechein
3500	299.9493	17.6	96.2	Ferulic [illegible]
4400	301.6803	14.0	42	Ellagic [illegible]
5000	302.8343	18.7	112.6	Gallic acid
5500	347.1293	13.9	39.9	4-Amino- benzoic acid
6000	411.4243	11.7	7.3	Coumarin
6500	475.7193			
7000	460.0143			

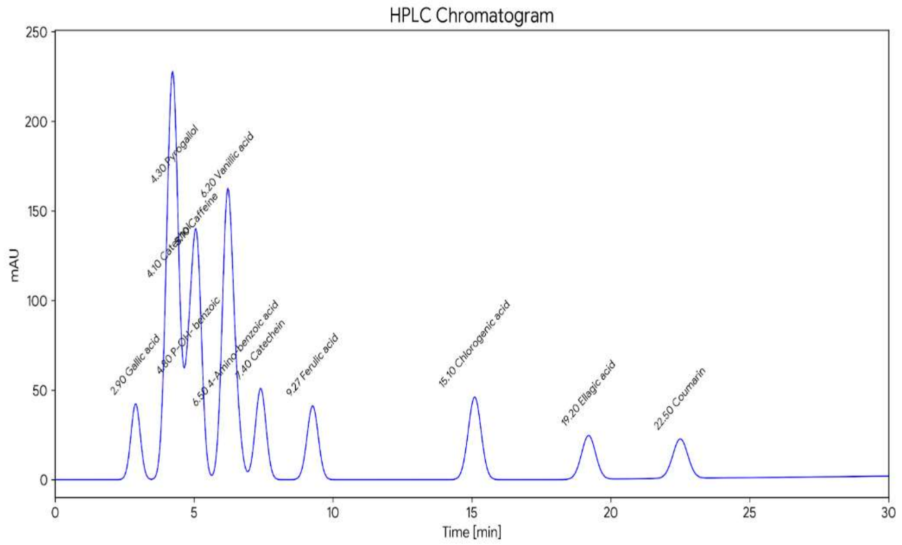

DFSB

Phenols µg/100 g	RT (min)	Area	Amount (µg/100 mL)
**Gallic acid**	2.9	12.4	17.2
**Catechol**	4.1	19.8	128.27
**Pyrogallol**	4.3	37.0	388.5
**P-OH- benzoic**	4.8	15.7	67.7
**Caffeine**	5.1	37.0	387.6
**Vanillic acid**	6.2	37.0	387.8
**4-Amino-benzoic acid**	6.5	12.1	12.7
**Catechein**	7.4	16.0	71.7
**Ferulic acid**	9.27	14.3	46.2
**Chlorogenic acid**	15.1	16.8	84.3
**Ellagic acid**	19.2	13.1	27.52
**Coumarin**	22.5	11.5	4.5

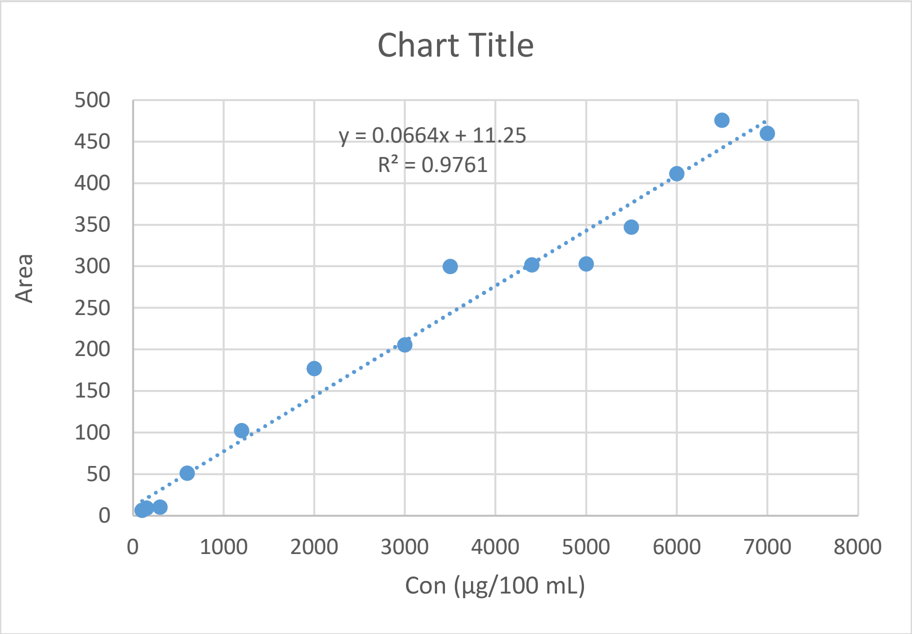

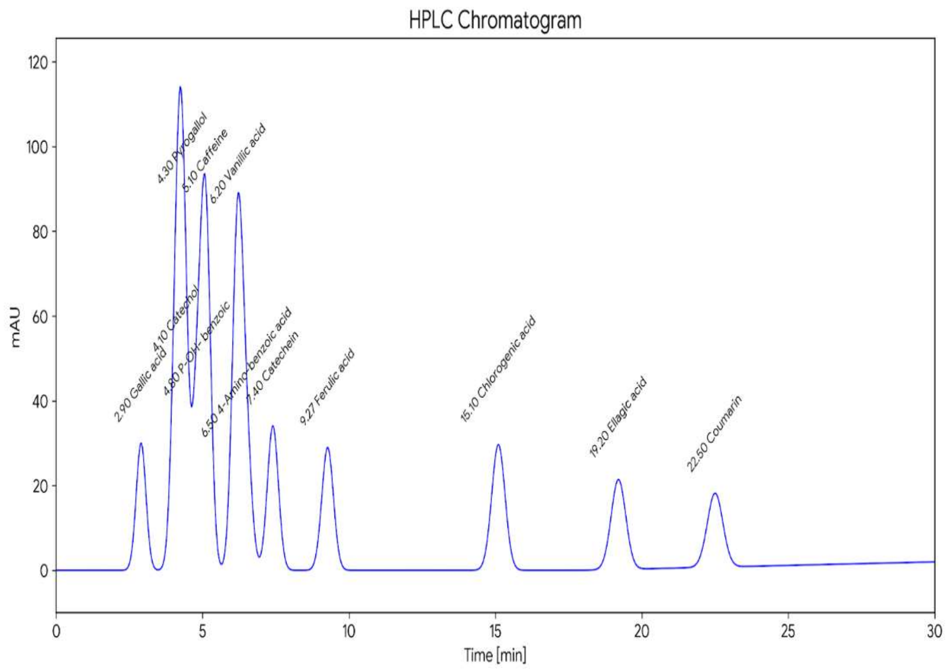

**Standard**		**Experiment**		
Con (µg/10(	Area	**Area**	**Amount (µg/100 mL)**	
100	6.6033	37.0	387.8	Vanillic acid
150	9.1728	37.0	388.5	Pyrogallol
300	10.4613	37.0	387.6	Caffeine
600	51.0383	19.8	128.27	Catechol
1200	102.1923	16.8	84.3	Chlorogenic acid
2000	177.0643	15.7	67.7	P-OH-benzoic
3000	205.6543	16.0	71.7	Catechein
3500	299.9493	14.3	46.2	Ferulic acid
4400	301.6803	13.1	27.52	Ellagic acid
5000	302.8343	12.4	17.2	Gallic acid
5500	347.1293	12.1	12.7	4-Amino-benzoic acid
6000	411.4243	11.5	4.5	Coumarin
6500	475.7193			
7000	460.0143			
